# An innovative EEG-based emotion recognition using a single channel-specific feature from the brain rhythm code method

**DOI:** 10.3389/fnins.2023.1221512

**Published:** 2023-07-20

**Authors:** Jia Wen Li, Di Lin, Yan Che, Ju Jian Lv, Rong Jun Chen, Lei Jun Wang, Xian Xian Zeng, Jin Chang Ren, Hui Min Zhao, Xu Lu

**Affiliations:** ^1^School of Computer Science, Guangdong Polytechnic Normal University, Guangzhou, China; ^2^Engineering Research Center of Big Data Application in Private Health Medicine, Fujian Province University, Putian, China; ^3^Hubei Province Key Laboratory of Intelligent Information Processing and Real-Time Industrial System, Wuhan University of Science and Technology, Wuhan, China; ^4^Guangxi Key Lab of Multi-Source Information Mining and Security, Guangxi Normal University, Guilin, China; ^5^New Engineering Industry College, Putian University, Putian, China; ^6^National Subsea Centre, Robert Gordon University, Aberdeen, United Kingdom

**Keywords:** electroencephalography (EEG), emotion recognition, brain rhythm, feature selection, machine learning

## Abstract

**Introduction:**

Efficiently recognizing emotions is a critical pursuit in brain–computer interface (BCI), as it has many applications for intelligent healthcare services. In this work, an innovative approach inspired by the genetic code in bioinformatics, which utilizes brain rhythm code features consisting of δ, θ, α, β, or γ, is proposed for electroencephalography (EEG)-based emotion recognition.

**Methods:**

These features are first extracted from the sequencing technique. After evaluating them using four conventional machine learning classifiers, an optimal channel-specific feature that produces the highest accuracy in each emotional case is identified, so emotion recognition through minimal data is realized. By doing so, the complexity of emotion recognition can be significantly reduced, making it more achievable for practical hardware setups.

**Results:**

The best classification accuracies achieved for the DEAP and MAHNOB datasets range from 83–92%, and for the SEED dataset, it is 78%. The experimental results are impressive, considering the minimal data employed. Further investigation of the optimal features shows that their representative channels are primarily on the frontal region, and associated rhythmic characteristics are typical of multiple kinds. Additionally, individual differences are found, as the optimal feature varies with subjects.

**Discussion:**

Compared to previous studies, this work provides insights into designing portable devices, as only one electrode is appropriate to generate satisfactory performances. Consequently, it would advance the understanding of brain rhythms, which offers an innovative solution for classifying EEG signals in diverse BCI applications, including emotion recognition.

## 1. Introduction

**Figure 1:**
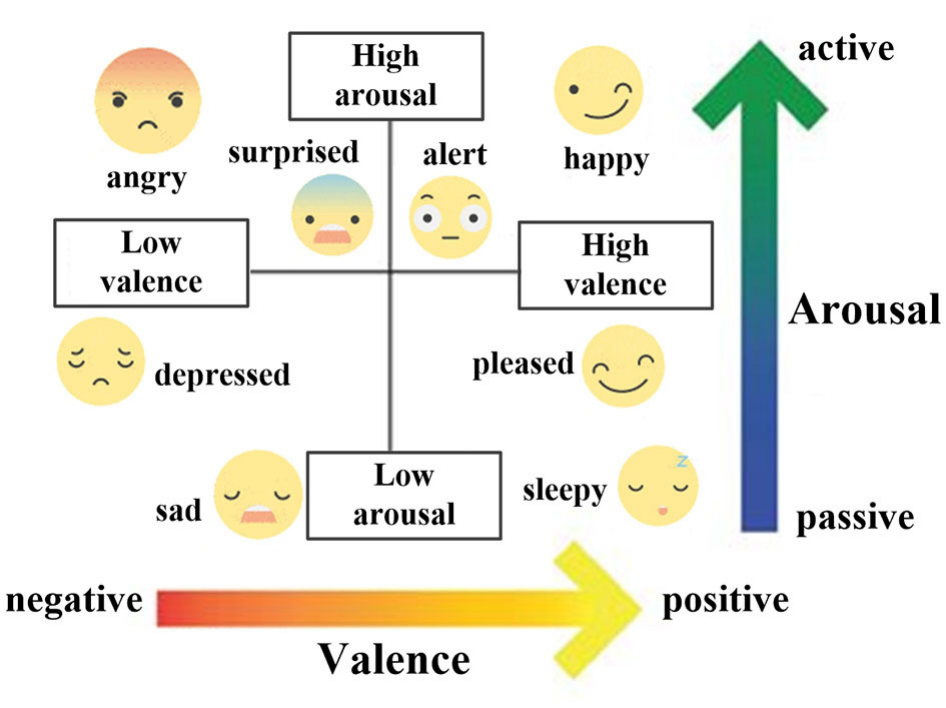
Two-dimensional Russell's emotional model based on arousal and valence.

Emotions are vital indicators of the psycho-physiological state of humans and can greatly help in improving intelligent healthcare services (Li C. et al., 2022). Therefore, developing approaches for the automatic recognition of emotions become a critical pursuit in brain–computer interface (BCI). To achieve this goal, an emotional model is necessary, and psychologists have traditionally modeled emotions in two ways. One is to categorize emotions into discrete types and use word descriptions such as happiness, anger, fear, disgust, sadness, and surprise to label them (Ekman, 1992). Nevertheless, this model may not accurately reflect the complex relationships between emotions, such as the connection between liking and pleasure. In addition, the same description can convey different intensities of emotion. Hence, a quantitative space model that adopts multiple dimensions to present emotions is more suitable. In this regard, Russell's emotional model (Russell, 1980), as shown in Figure 1, presents emotions in a two-dimensional space consisting of arousal and valence. Although similar emotions may have overlapping descriptions, they can be measured by intensities according to arousal and valence. So, it enables the quantification and differentiation of emotions, providing a universal model for emotion recognition (Nawaz et al., 2020).

Emotion recognition is currently accomplished through a range of sensing mechanisms that mainly include non-physiological and physiological signals. Facial expressions (Soleymani et al., 2016), speech (Li et al., 2023), text (Bharti et al., 2022), and body gestures (Sun et al., 2018) are classical non-physiological signals that capture the external manifestations of emotions. Physiological signals reflect the changes that occur within the body as a response to emotional tendencies, such as electromyography (EMG) (Kulke et al., 2020), electrooculography (EOG) (Lim et al., 2020), and electrocardiography (ECG) (Rinella et al., 2022). Usually, physiological signals are spontaneously generated by the body, so it is commonly believed that they objectively reflect emotions and offer higher reliability than non-physiological signals.

From a neuroscience perspective, emotions arise from the inner reactions associated with the central nervous system (CNS), indicating that the brain acts as the source of emotions (Adolphs, 2017). Therefore, electroencephalography (EEG), the physiological signals from the brain, is trusted to be appropriate for emotion recognition (Wu et al., 2023). Furthermore, with the development of portable BCI, EEG-based emotion recognition is considered an efficient solution for affective computing. To this end, several critical operations are necessary, such as utilizing proper signal processing methods to extract reliable features, selecting optimal features or representative channels to achieve satisfactory results, and applying suitable machine learning classifiers to facilitate classification. Thus, it is essential to investigate each step for remarkable emotion recognition results.

Various signal processing methods have been employed in the past to extract features from the EEG signals and then used for emotion recognition. The time-domain features are extracted through statistical measures, such as mean, standard deviation, and root mean square (RMS) (Chakladar and Chakraborty, 2018). The frequency-domain features rely on spectral characteristics, such as spectral envelope, power spectral density (PSD), and higher-order spectra (HOS) (Yuvaraj et al., 2014). Moreover, the time–frequency analysis (TFA) provides additional information on dynamic variations of EEG across both time and frequency domains for extracting features. Its classical techniques include wavelet transform (WT) (Gupta et al., 2018), Hilbert–Huang transform (HHT) (Chang et al., 2022), and Wigner–Ville distribution (WVD) (Alazrai et al., 2018). Finally, other features, such as entropy (approximate entropy, Kolmogorov–Sinai entropy, and differential entropy) that quantify the irregularity of EEG (Nalwaya et al., 2022), connectivity (differential asymmetry, rational asymmetry, and magnitude-squared coherence) that reveal the asymmetry of EEG (Li et al., 2020), and chaos (Lyapunov exponent, correlation dimension, and embedding dimension) that indicate the complexity of EEG (Li et al., 2018), can also be applied in this field.

Generally, several frequency sub-bands present in the EEG signals, i.e., five brain rhythms: δ (0–4 Hz), θ (4–8 Hz), α (8–13 Hz), β (13–30 Hz), and γ (30–50 Hz), offer insights into the characteristics of neuronal activities in the brain (Choi and Kang, 2014), which indicates each has its generation mechanism and is indicative of specific emotional states. For instance, δ power tends to increase during the transition from exciting to calming (Lee et al., 2020), while an increase in θ power over left frontocentral sites is associated with joy (Reuderink et al., 2013). α power owns an inverse relationship with the level of arousal (Koelstra et al., 2012), and an increase in β power over the right temporal region is linked to passion, hope, and gratitude (Zheng et al., 2017). Finally, an increase in γ power is related to sadness, depression, and pain (Fitzgerald and Watson, 2018). As a result, brain rhythms provide vital indicators for assessing emotions, and that is why they have been extensively adopted in recent EEG-based emotion recognition (Sarma and Barma, 2021).

EEG systems are typically multichannel in nature, resulting in numerous features extracted from multichannel signals (Taran and Bajaj, 2019). It is widely recognized that employing a large number of channels or features in emotion recognition poses practical limitations in real-life scenarios. Such limitations include prolonged experimental setup times, subject discomfort, and increased computational complexity associated with handling multichannel EEG recordings (Athavipach et al., 2019). Given these constraints, there is considerable value in developing a portable emotion-aware BCI that focuses on an optimal feature from the representative channel. By doing so, the complexity of emotion recognition can be significantly reduced, making it more achievable for hardware setups. This approach not only streamlines the experimental process but also reduces the burdens on the subjects and the computational resources. Hence, feature selection is particularly vital in EEG-based emotion recognition (Lin et al., 2023). For this purpose, it is necessary to investigate various features with different machine learning classifiers. From the method evaluation, the optimal feature that produces satisfactory accuracy can be selected accordingly.

Several studies have been conducted to realize EEG-based emotion recognition using optimal features from the representative channels. Menezes et al. (2017) extracted the PSDs, statistical features, and high-order crossings (HOCs) from four channels on the frontal region (FP1, FP2, F3, and F4), then used the support vector machine (SVM) to obtain classification accuracies of 67.1% and 88.9% for arousal and valence. Javidan et al. (2021) investigated the features from time, frequency, and coherence domains, along with support vector regression (SVR), linear regression, and multilayer perceptron (MLP) acted as the classifiers. The results demonstrated that using the SVR, the magnitude-squared coherence estimate (MSCE) features from F7 and F8 channels produce an accuracy of 67.5% on valence. Anuragi et al. (2022) employed Fourier–Bessel series expansion-based empirical wavelet transform (FBSE-EWT) to extract PSD features from the frontal region, and then k-nearest neighborhood (k-NN), artificial neural network (ANN), and ensemble bagged tree were deployed. The results presented that neighborhood component analysis (NCA) helps to select the optimal features that provide the accuracies of 84.3% and 83.9% to classify arousal and valence, and 78.1% to recognize negative, neutral, and positive states. Kannadasan et al. (2023) proposed the differential evolution-based feature selection (DEFS) method that can choose the optimal features. The results displayed that the features of θ, α, and β from AF4, FP2, F7, FZ, FC1, FC2, CP2, CP5, CP6, P7, P8, PZ, and T7 channels realize an arousal accuracy of 74.23%, and the features of α and β from AF3, F3, F4, F8, FZ, FC6, CZ, CP2, P4, P8, and OZ channels achieve a valence accuracy of 73.60%.

Previous studies have mostly focused on features from coherence, entropy, statistical measures, and PSD. However, the temporal dynamics of brain rhythms during emotional states have not been fully considered. To this end, an innovative approach that concentrates on the features derived from temporal dynamics of brain rhythms is proposed, which provides a new perspective on analyzing rhythmic variations of EEG for establishing an emotion recognition method. Moreover, the single optimal feature for each case can be identified by considering the highest accuracy among all features. This solution is more convenient to the subjects as it only requires one electrode or sensor, improving the portability of BCI accordingly. In addition, the individual differences in emotional responses can be investigated based on the optimal features found, providing insights into the mechanisms underlying emotional processing in the brain. Now, taking inspiration from bioinformatics, the genetic codes consisting of three bases (A, G, C, or T) can be extracted and utilized for classifying amino acids or polypeptides (Chin, 2017). Following this way, brain rhythms can be presented in a sequential format based on time-related variations, allowing for extracting code features for emotion recognition. Thus, this study designs the use of brain rhythm codes from three bases (δ, θ, α, β, or γ) of the sequences generated by the brain rhythm sequencing (BRS) technique previously proposed for seizure detection (Li et al., 2019). After that, four conventional machine learning classifiers, including k-NN, SVM, linear discriminant analysis (LDA), and logistic regression (LR), are evaluated for those extracted code features, with the aim of identifying the single optimal channel-specific feature and a suitable classifier for accomplishing satisfactory emotion recognition accuracies through the minimal data. Specifically, the following contributions are obtained in this study:

■ The capability of BRS to extract code features that reveal consecutive time-related variations of brain rhythms is proposed, which offers an innovative way to investigate the relationship between rhythmic occurrences and emotional variations in EEG-based emotion recognition.
■ Based on the extracted brain rhythm codes, four classifiers (k-NN, SVM, LDA, and LR) are evaluated to determine the single optimal channel-specific feature that produces the highest accuracy for emotion recognition. This feature selection helpfully simplifies the emotion-aware BCI device, making it available for practical applications while maintaining satisfactory accuracy.
■ The method performances are validated on three emotional datasets: DEAP (Koelstra et al., 2012), SEED (Zheng and Lu, 2015), and MAHNOB (Soleymani et al., 2012). Such validations provide insights into the effects of brain rhythms and channels on emotion recognition, as well as individual differences. Meanwhile, the cross-corpus results also demonstrate the effectiveness of the proposed method under various conditions.

**Figure 2:**
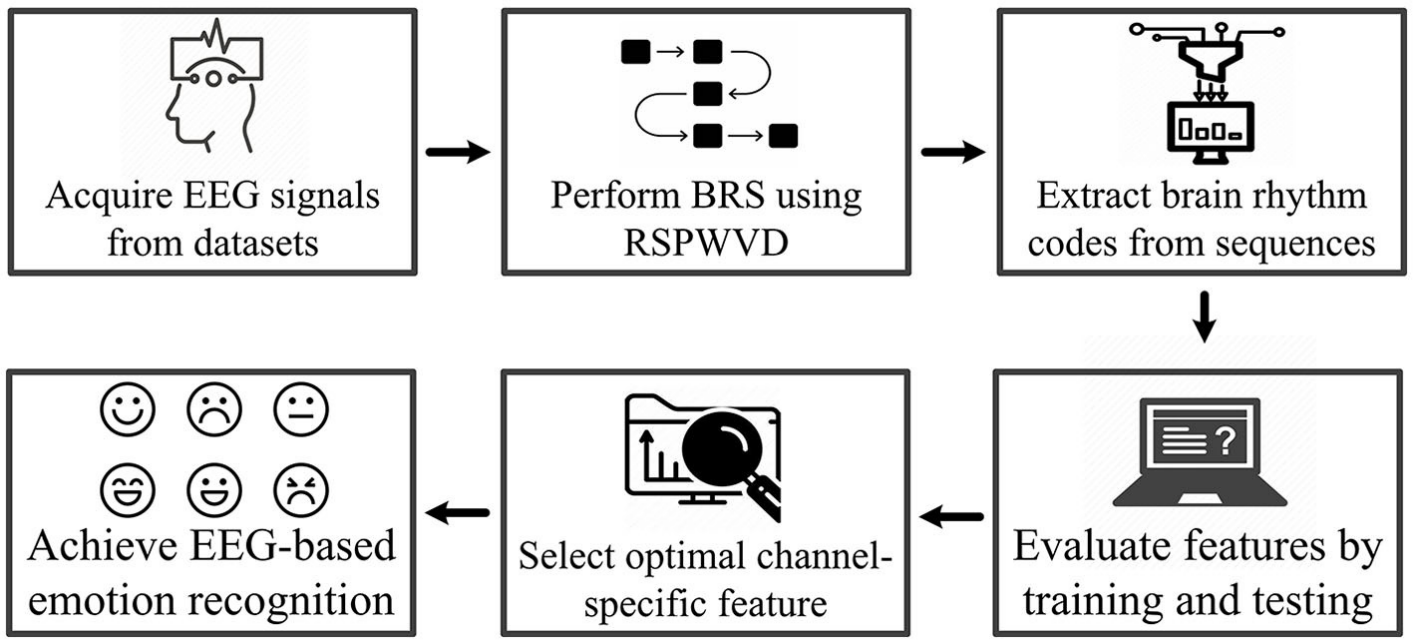
The system workflow of this work.

The system workflow is illustrated in Figure 2. First, the EEG signals from three emotional datasets are acquired, and the BRS technique is then performed by the reassigned smoothed pseudo-Wigner–Ville distribution (RSPWVD) to generate brain rhythm sequences that indicate the vital time–frequency characteristics of EEG. Next, the code features consisting of three rhythmic bases (i.e., δ*δδ*, δ*δθ*, ..., γ*γγ*) are extracted from each generated sequence, resulting in a total of 125 features per sequence. Subsequently, all of them are evaluated through the leave-one-trial-out cross-validation (LOTO-CV) training and testing by applying four classifiers, and according to the highest accuracy on each case, the single optimal channel-specific feature is selected. Finally, this optimal feature is used to achieve recognition and explore individual differences. For better reading comprehension, Table 1 summarizes the definition of acronyms in this study.

**Table 1:** Definition of acronyms.

**Acronym**	**Description**	**Acronym**	**Description**
A	Arousal	V	Valence
HA	High arousal	HV	High valence
LA	Low arousal	LV	Low valence
DE	Differential entropy	LR	logistic regression
k-NN	k-nearest neighborhood	ToC	Third-order cumulant
ANN	Artificial neural network	BCI	Brain–computer interface
BRS	Brain rhythm sequencing	CNS	Central nervous system
CPU	Central processing unit	DBN	Deep belief network
DWT	Discrete wavelet transform	ECG	Electrocardiography
EEG	Electroencephalography	EMD	Empirical mode decomposition
EMG	Electromyography	EOG	Electrooculography
HHT	Hilbert–Huang transform	HOC	High-order crossing
HOS	Higher-order spectra	IMF	Intrinsic mode function
LDA	Linear discriminant analysis	MLP	Multilayer perceptron
NCA	Neighborhood component analysis	PSD	Power spectral density
PSO	Particle swarm optimization	RAM	Random access memory
RBF	Radial basis function	RMS	Root mean square
SAM	Self-assessment manikin	SVM	Support vector machine
SVR	Support vector regression	TFA	Time–frequency analysis
WVD	Wigner–Ville distribution	DEFS	Differential evolution-based feature selection
HSLT	Hierarchical spatial learning transformer	LSTM	Long short-term memory
MSCE	Magnitude-squared coherence estimate	ANOVA	Analysis of variance
SPWVD	Smoothed pseudo-Wigner–Ville distribution	RSPWVD	Reassigned smoothed pseudo-Wigner–Ville distribution
LOTO-CV	Leave-one-trial-out cross-validation	FBSE-EWT	Fourier–Bessel series expansion-based empirical wavelet transform

The rest of this study is organized as follows: Section II presents the details of the three experimental datasets. Section III describes the proposed method that involves brain rhythm code feature extraction and machine learning-based classification. Section IV includes the experimental results with discussions and the comparative study. Finally, Section V summarizes this study.

## 2. Experiments

The first critical step in an EEG-based study is high-quality data acquisition. To ensure the reliability of this study, three well-established emotional datasets were analyzed: DEAP, SEED, and MAHNOB. These three datasets contained audio–visual stimuli (music videos and movie clips) that were presented to the subjects in their native language, and the gender ratio was balanced. In addition, the self-assessment manikin (SAM) ratings and emotional labels were incorporated into the datasets, serving as ground truth measures for emotion recognition tasks. Furthermore, EEG recordings were acquired using international standards to guarantee data quality. Usually, the raw recordings contained EOG artifacts, particularly in the frontal region. Such artifacts can impact the overall performance, necessitating their substantial reduction during the preprocessing stage. In this regard, the three datasets provided preprocessed data; so relatively, artifact-free EEG data were used in this study. Table 2 summarizes the information from three datasets, and more details are described in the subsequent sections.

**Table 2:** Three experimental datasets in this study.

	**DEAP (Koelstra et al., 2012)**	**SEED (Zheng and Lu, 2015)**	**MAHNOB (Soleymani et al., 2012)**
Number of subjects	32	15	24
Number of experimental trials	40	45	20
Time length of EEG data	1 min	4 min	34.9–117 s
Emotional labels	High/low arousal (A ≥ 5, A <5) High/low valence (V ≥ 5, V <5)	Negative, neutral, positive	High/low arousal (A ≥ 5, A <5) High/low valence (V ≥ 5, V <5)
Test groups	Group-A, Group-V (2 classes)	Group-NNP (3 classes)	Group-A, Group-V (2 classes)
Number of channels	32	62	32
Down-sampling rate of EEG	128 Hz	200 Hz	256 Hz

### 2.1. DEAP

The DEAP dataset employed 40 one-min music videos as stimuli to evoke emotional states, resulting in 40 experimental trials per subject, and a total of 32 subjects participated, with an average age of 27.19 ± 4.45 years. The EEG recordings were obtained by operating the 10–20 system with 32 scalp channels. Therefore, each subject's total data size was 40 trials × 32 channels × 60 s. The subjects performed SAM to rate each music video (1–9) in terms of arousal (A) and valence (V). As the threshold for high and low was 5 (Mohammadi et al., 2017), the emotional states were then categorized into two test groups for method evaluation, where Group-A contained high arousal (HA) and low arousal (LA), and Group-V included high valence (HV) and low valence (LV). Additionally, the EEG data were preprocessed by adopting an analog band-pass filter with a cutoff frequency of 0.01–100 Hz and down-sampled to 128 Hz.

### 2.2. SEED

The SEED dataset contained EEG signals from 15 subjects using 62 scalp channels, with an average age of 23.27 ± 2.37 years. Each of them participated in three experimental sessions on different days, while one session involved 15 movie clips that can evoke negative, neutral, or positive states. Moreover, to ensure balanced data acquisition, each emotion was evoked by five stimuli. Thus, emotion recognition was evaluated in Group-NNP, a three-classes task. Additionally, the EEG data were collected at a sampling rate of 1000 Hz, then down-sampled to 200 Hz, and band-pass filtered to 0.5–70 Hz for preprocessing.

### 2.3. MAHNOB

The MAHNOB dataset included EEG signals from 32 scalp channels, and a total of 30 subjects participated, with an average age of 26.06 ± 4.39 years. However, due to technical issues, data from 6 subjects were incomplete, leaving 24 subjects for analysis in this study. The experiment applied 20 movie clips (duration: 34.9–117 s) to elicit emotional states. After each clip, subjects rated their arousal and valence levels based on 1–9, similar to the SAM in the DEAP dataset. Hence, the resulting emotions were also labeled into two test groups (Group-A and Group-V) for method evaluation. Additionally, the EEG data were recorded at 1024 Hz, then down-sampled to 256 Hz for preprocessing.

## 3. Methodology

The proposed method includes three critical steps. First, the RSPWVD is utilized to extract the characteristics hidden in the time–frequency plane, which assists in achieving BRS from EEG. Next, brain rhythm code features are extracted from each generated sequence, as one EEG channel corresponds to one rhythm sequence, resulting in a vast number of features. Finally, the classification method is established by those extracted code features, and four conventional classifiers are adopted for training and testing through LOTO-CV. Further details on the above steps are described in the following subsections.

### 3.1. BRS based on RSPWVD

EEG recordings are highly sensitive to variations in brain states, and changes in brain rhythms occur dynamically as subjects transition from one emotional state to another. Brain rhythms are, therefore, considered vital indicators of emotional states. To obtain insights into EEG signals, a sequence that represents the temporal dynamics of brain rhythms is helpful. To this end, an appropriate signal processing method that generates precise time–frequency characteristics, especially the details regarding the instantaneous signal powers at both time and frequency domains, is necessary.

There are several classical techniques for processing EEG signals, such as discrete wavelet transform (DWT) and empirical mode decomposition (EMD). By applying DWT, the EEG signals can be decomposed into distinct levels that correspond to diverse frequency sub-bands. For instance, D5 represents 4–8 Hz, D4 represents 8–16 Hz, D3 represents 16–32 Hz, D2 represents 32–64 Hz, and D1 represents 64–128 Hz (Mohammadi et al., 2017). However, it is found that these decompositions are recursive and do not precisely align with the designated ranges of the five brain rhythms. Similarly, EMD, a method adept at extracting intrinsic mode functions (IMFs) from EEG, faces challenges in associating such IMFs with the five brain rhythms. For example, IMF1 captures components of EEG with the highest frequency characteristics, embodying both β and γ properties (Zhuang et al., 2017). Additionally, EMD suffers from undesirable end effects that render both ends of the data meaningless, thereby affecting its applicability in power quality analysis (Wang et al., 2019). In this regard, WVD offers a high-resolution spectral analysis and is well-suited for extracting features from multicomponent signals (Barma et al., 2016). It enables the acquisition of time–frequency characteristics, particularly the instantaneous power distributions within the five brain rhythms of EEG. WVD is mathematically expressed as:


(1) $$\begin{array}{l} W_{x}\left(t,\omega \right)\;=\;\overset{+\infty }{\underset{-\infty }{\int }}x\left(t+\frac{\tau }{2}\right)x^{*}\left(t-\frac{\tau }{2}\right)e^{-j\omega \tau }d\tau \end{array}$$

where *x*(*t*) represents the input signals, *t* and ω are time and frequency, and ^*^ denotes the complex conjugate.

Nonetheless, WVD has a cross-term issue that incurs an inaccurate assessment of frequency within a short time interval, meaning that the artifact can falsely show the power localization of a component in the time–frequency plane. To address it, a smoothing version of the WVD is deployed by applying a separable smoothing window function in time and frequency domains. This progressive and independent approach is proper to extract the time-varying information of EEG accordingly. Such a way is defined as smoothed pseudo-Wigner–Ville distribution (SPWVD):


(2) $$\begin{array}{l} \;SPW_{x}\left(t,w\right)\;=\\ \overset{+\infty }{\underset{-\infty }{\int }}h\left(\tau \right)\overset{+\infty }{\underset{-\infty }{\int }}g\left(s-t\right)x\left(s+\frac{\tau }{2}\right)x^{*}\left(s-\frac{\tau }{2}\right)e^{-j\omega \tau }dsd\tau \end{array}$$

where, *g*(*t*) and *h*(*t*) are the smoothing windows employed for time and frequency, respectively.

Furthermore, the reassignment method is effective in improving the time–frequency concentration properties of the auto-terms of signal components, making it advantageous to assess the instantaneous power distribution with high resolution, so it can be incorporated into the SPWVD and generates the RSPWVD. This procedure reassigns each value of the SPWVD at any point (*t*, ω) to another point ($\hat{t},\hat{\omega }$), the center of gravity of the power distribution around (*t*, ω), within the time–frequency domain, as presented below:


(3) $$\begin{array}{l} \;SPW_{x}^{\left(r\right)}\left(t^{\prime }\omega^{\prime };g,\;h\right)\;=\\ \overset{+\infty }{\underset{-\infty }{\int }}\overset{+\infty }{\underset{-\infty }{\int }}SPW_{x}\left(t\;\omega ;g,\;h\right)\delta \left(t^{\prime }-\hat{t}\left(x;\;t,\omega \right)\right)\delta \left(\omega^{\prime }-\hat{\omega }\left(x;\;t,\omega \right)\right)dtd\omega \end{array}$$

where:


(4) $$\begin{array}{lll} \hat{t}\left(x;\;t,\;\omega \right)\; & = & \;t-\frac{SPW_{x}\left(t,\;\omega ;\;\tau_{g},\;h\right)}{2\pi SPW_{x}\left(t,\;\omega ;\;g,\;h\right)} \end{array}$$

(5) $$\begin{array}{lll} \hat{\omega }\left(x;\;t,\;\omega \right)\; & = & \;\omega +j\frac{SPW_{x}\left(t,\;\omega ;\;g,\;D_{h}\right)}{2\pi SPW_{x}\left(t,\;\omega ;\;g,\;h\right)} \end{array}$$

with τ_*g*_ = *tg(t)* and *D*_*h*_*(t)* = *dh(t)/dt*.

**Figure 3:**
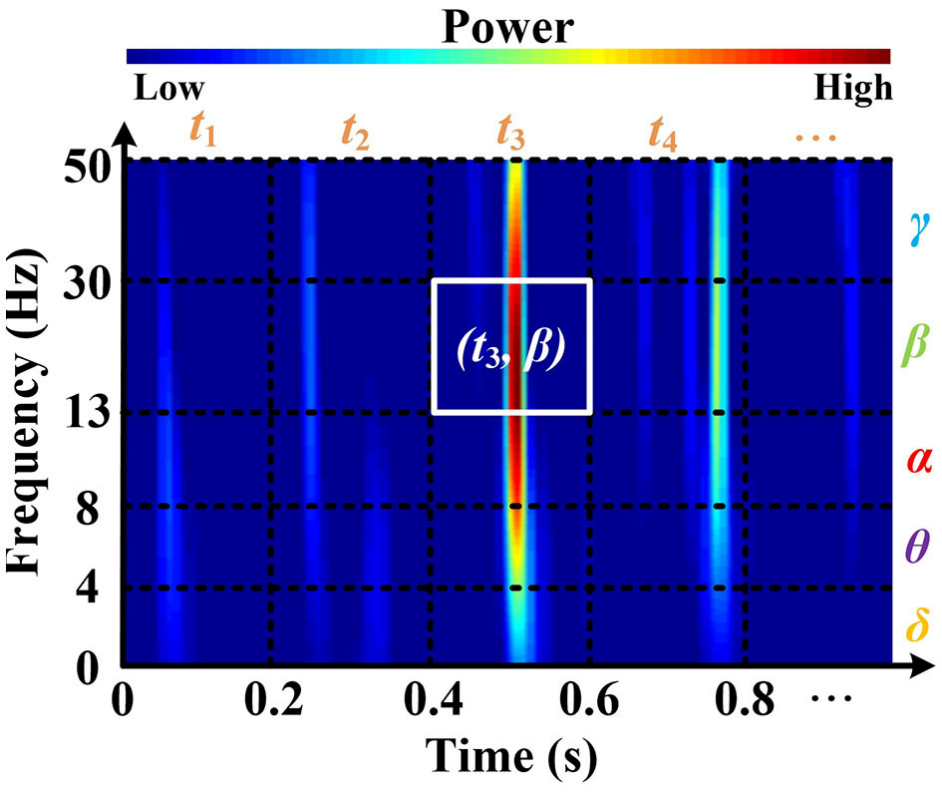
The time-frequency plane is generated by applying the RSPWVD method, which can appropriately provide instantaneous power distributions with time-varying information from EEG. Data is from DEAP, Subject S8, FP1 channel.

**Figure 4:**
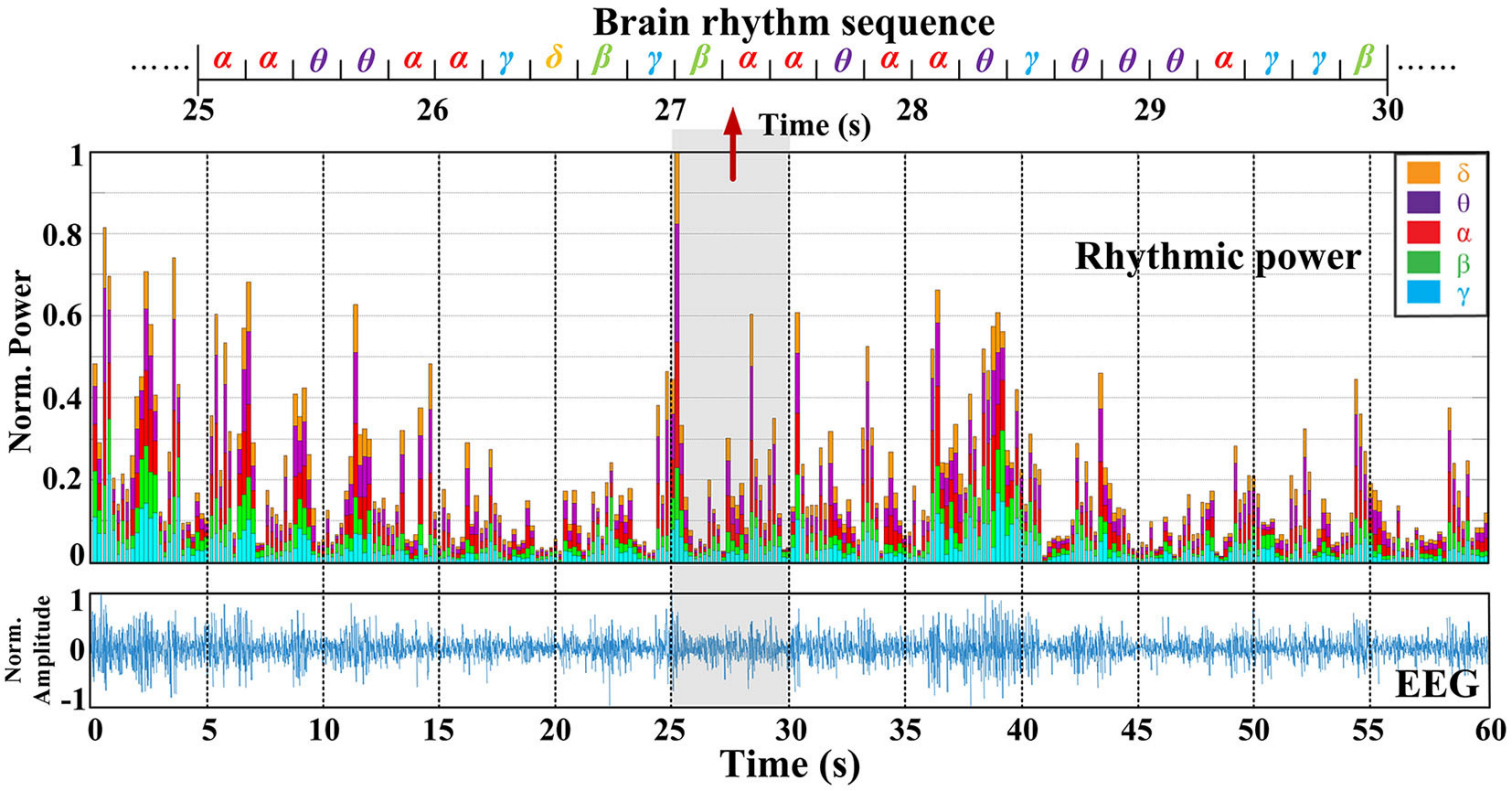
The sequential format of EEG is produced by the dominant brain rhythm having the maximal instantaneous power at each timestamp within 0.2 seconds. Data is the same as assessed in Figure 3.

Here, δ(.) denotes the Dirac impulse. In particular, the RSPVWD exhibits time and frequency shift-invariant and respects the energy conservation property, which not only eliminates the cross-terms in both time and frequency domains but also makes the instantaneous power distributions properly represented in the five brain rhythms on their time-related localizations. Figure 3 depicts a time–frequency plane from an EEG signal (DEAP, Subject S8, FP1 channel) generated by the RSPWVD, which provides a clear visualization of the instantaneous power distributions of the signal with time-varying information. Then, to express such characteristics as a time-related sequence, the time axis is further separated into different timestamps (i.e., *t*_1_, *t*_2_, *t*_3_, *t*_4_, …,) based on 0.2 s interval, referenced by the average reaction time of neurons (Rey et al., 2014). The reason is that electrical responses of neuronal firings can be measured in the EEG, and an average reaction time of neurons implies a duration that appears those firings, so the electrical responses of the EEG signals can be studied within approximately 0.2 s duration correspondingly. Moreover, as brain rhythms are vital components in the sequence, the frequency axis is divided into five respective parts (δ, θ, α, β, and γ). After that, it is essential to identify the dominant rhythm at each timestamp. To this end, instantaneous power, which has been demonstrated to exhibit a close relationship with emotion recognition, is utilized. For instance, in Figure 3, the β power at *t*_3_ is calculated by the average of all powers located in the white box that corresponds to the timestamp *t*_3_ and β rhythm. By repeating this process, five rhythmic powers located at diverse timestamps can be obtained in detail, as illustrated in the middle of Figure 4. Finally, a dominant rhythm having the maximal instantaneous power at each timestamp is acquired for sequencing. This technique is called BRS. A portion of the generated sequence (25–30 s) is depicted at the top of Figure 4, where it is from an EEG signal (DEAP, Subject S8, FP1 channel, the same data assessed in Figure 3) presented at the bottom of Figure 4.

### 3.2. Code feature extraction

**Figure 5:**
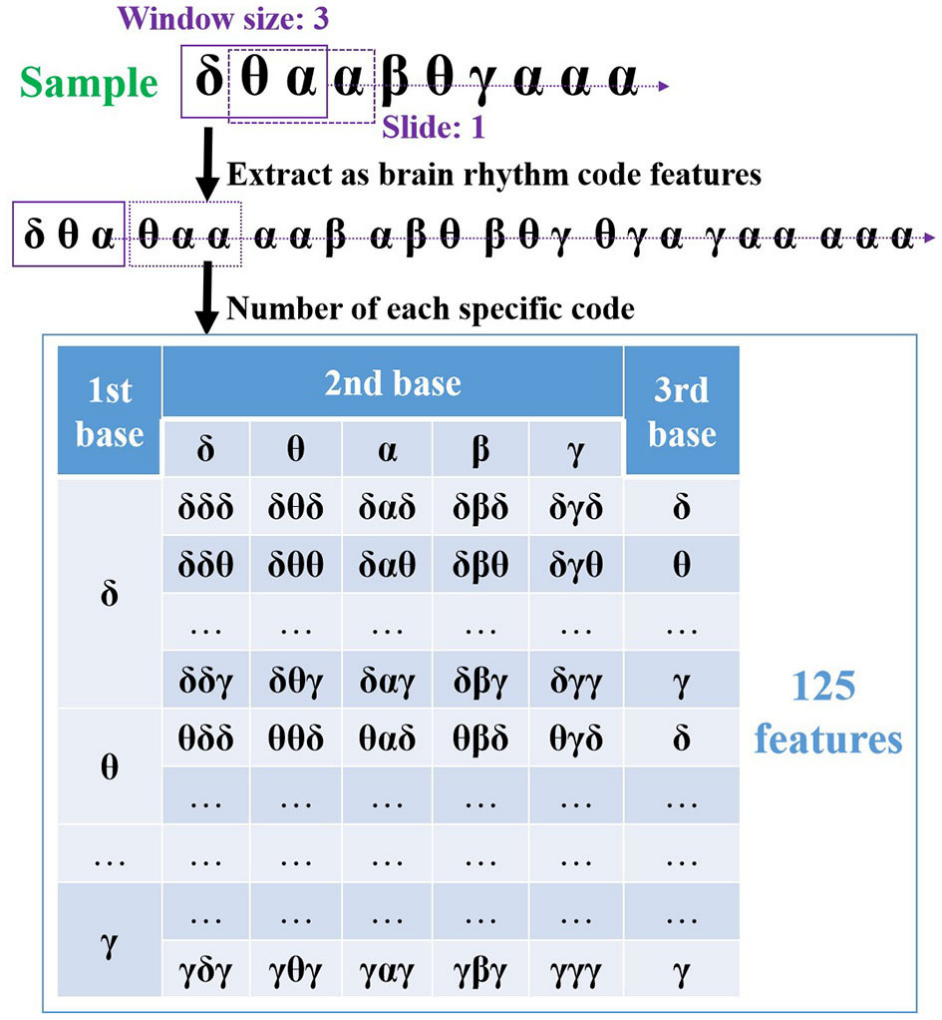
The proposed code features extraction based on three rhythmic bases with sliding one step size from the generated sequence data.

As an internal reaction to external stimuli, emotion combines the thoughts, feelings, and actions controlled by the brain. In this regard, an EEG system records the electrical responses from the brain through multichannel over different regions, and when the rhythm sequence is produced for each channel, such sequences correlate with various special regions. Therefore, toward emotion recognition, it is critical to extract useful channel-specific features from them. Considering that the sequence data enclose the temporal dynamics of dominant rhythms, the rhythmic features are considered in the feature extraction, aiming to disclose the chronological variations of rhythms presented in the generated sequence. Hence, inspired by the genetic code derived from three bases (A, G, C, or T) in bioinformatics, the code features based on three rhythmic bases (i.e., δ*δδ*, δ*δθ*, ..., γ*γγ*) are extracted from each generated sequence. Such features show the patterns of brain rhythms along the time scale, usefully revealing how they vary with emotional variations. As a result, it provides an innovative manner to study the connection between EEG brain rhythms and emotions. For illustration, Figure 5 outlines a sample of code feature extraction.

In Figure 5, a window size of three rhythmic bases is applied and slid along the sequence with one step size at first. Hence, eight code features are extracted from this sequence correspondingly. The number of each code is then obtained by considering its appearances, which can reflect the variations under different emotions. For instance, what appears to code feature δ*θα* when the valence decreases or increases? Finally, as these code features contain three bases and there are five brain rhythms in the EEG study, a total of 125 features can be extracted from one sequence (channel). Please note that several code features may not appear in the generated sequence, resulting in its value of 0. Therefore, if a system records from 32 channels, a group of 4000 code features is extracted by the proposed method, and each of them contains channel and rhythm details, i.e., channel-specific information.

Moreover, the EEG time length affects feature extraction, as a longer time length results in more occurrences, while a shorter one makes fewer. To solve it, the data in this study should be with the same time length so that all extracted code features are fair for method evaluation. In this regard, it is necessary to consider a suitable period for emotion recognition. Previously, Jatupaiboon et al. (2013) achieved emotion recognition in terms of arousal and valence through the first 30 s, the last 30 s, and the whole 60 s of EEG. The results claimed that the last 30 s of data produce the highest accuracy, suggesting this period is sufficient for assessing emotions and capturing relevant patterns. In another study, Bhattacharyya et al. (2021) mentioned that to avoid mood swings of emotion recognition, the last 30 s of EEG from each trial is preferred. Based on these findings, the code feature extraction is performed on the last 30 s of data from the three emotional datasets, and then applied to conventional machine learning classifiers for training and testing, as described in the next step.

### 3.3. Classification method

Recent studies (Ji and Dong, 2022; Li Z. et al., 2022; Xiao et al., 2022) have highlighted the neural networks in emotion recognition. These networks are capable of learning high-level features from raw EEG data in an incremental manner, which eliminates the requirement for feature extraction. Although neural networks have demonstrated high accuracies in emotion recognition, they are better suited for larger datasets, as the high dimensionality of the data enhances the network's training. If the dataset is smaller, neural networks may encounter challenges. Meanwhile, when considering individual differences in emotion recognition, it may be more appropriate to establish a classification method based on subject-dependent effects. In addition, in this study, the feature size may not be large enough to train a robust neural network model. For example, in the DEAP dataset, 40 trials are provided, resulting in an extraction of 40 code features from the same channel on each subject. In such scenarios, conventional machine learning classifiers that can establish the model based on a small feature size are proper. As a result, k-NN, SVM, LDA, and LR are applied to evaluate the extracted code features and established the classification method.

First, concerning the k-NN, it stores all the training data and classifies testing data into the most proper class based on a distance *k* function employing a similarity measure. For instance, if *k* is 3, the nearest three neighbors of the testing data are obtained, and then the classification is achieved by the majority vote of such neighbors. Usually, *k* closes to the square root of the number of training data and is preferred to be a small positive odd integer. Based on that, *k* is set to 5, 5, and 3 for the DEAP, SEED, and MAHNOB, respectively.

Second, regarding the SVM, the standard SVM classifier is applied to the two-classes task (Group-A and Group-V). In SVM, the kernel function is a vital component that transforms the input data into a higher dimensional feature space, where it becomes easier to find a linear separating hyperplane, indicating that different kernel functions can enclose various effects on the decision boundary and classification performance. Hence, based on the DEAP dataset, a preliminary assessment has been performed to test the performances through four commonly used kernel functions, including the linear kernel, polynomial kernel, radial basis function (RBF) kernel, and sigmoid kernel. The testing results reveal that the classification accuracy using the RBF kernel is the best among the four, and thus, for all two-classes tasks in this study, the RBF kernel is included in the SVM classifier. Furthermore, for the three-classes task (Group-NNP), the SVM classifier is extended using the one-vs.-one (OVO) approach. In detail, a binary classifier is trained for each pair of classes initially, so three binary classifiers are trained: negative vs. neutral, neutral vs. positive, and negative vs. positive. Then, each binary classifier is trained on a subset of the data that contains only the samples from the two classes it is distinguishing. When classifying a new sample (testing data), each binary classifier predicts the class label, and the class with the majority of votes among all binary classifiers is assigned as the final predicted class, so the SVM can conduct the three-classes task through the OVO approach.

Third, LDA estimates the class means and covariances from the training data, uses these estimates to compute the class-conditional densities, and combines them with prior probabilities to calculate the posterior probabilities. The linear decision boundary is determined by finding the line that maximizes the separation between the classes based on the computed posterior probabilities. Therefore, using this linear decision boundary, LDA can classify new samples based on the feature values. In this regard, there are no specific parameters that need to be set, as LDA directly calculates the optimal discriminant vectors through class means and covariances. The most vital consideration is that the training data should provide the known class labels for the calculations. In addition, toward the three-classes task, the OVO approach is also required in the LDA.

Fourth, as for the LR, multinomial logistic regression is adopted, which can handle binary and multiple classification tasks. This algorithm employs the softmax function to model the probabilities of each class between 0 and 1, and the class with the highest probability is chosen as the predicted class. Hence, the training data are used to train the LR model directly, while the testing data are applied for evaluation following the outcomes of predicted probabilities. Again, the key is the training data should provide the known class labels.

More importantly, in machine learning, when dealing with a sufficient amount of data, it is common to randomly divide the evaluated features into three parts: training data, validation data, and testing data. However, due to the limited sample size of each extracted code feature, especially in the case of the MAHNOB dataset, this approach is not feasible. In this regard, conventional feature selection or dimensionality reduction methods may not perform appropriately due to insufficient data for training and validation. To address the challenge of insufficient size, LOTO-CV is employed, which particularly suits scenarios where the data size is small, and eliminates the need for a separate validation set by utilizing each trial as testing data once. The methodology involves systematically leaving out one trial at a time, training the model on the remaining trials, and evaluating its performance on the left-out trial. This process simulates a real-life scenario where the model is applied to new trials, thereby providing a more realistic estimate of its effectiveness. Additionally, LOTO-CV reduces bias as it utilizes all available data for training and validation, ensuring that the performance is not skewed by a specific partitioning of the data. Based on that, to determine the optimal feature, the same feature is split into the training data and testing data. Subsequently, LOTO-CV is performed, and classification accuracy is evaluated. Please note that for DEAP, MAHNOB, and SEED, the size of training data is 39, 19, and 44, respectively, and the size of testing data in all cases is 1, each time with different trials used for training and testing. By comparing the results across different features, the one that yields the highest accuracy can be selected and denoted as the optimal feature. Consequently, this manner allows for the identification of features that consistently contribute to accurate classification across different trial combinations, which can avoid overfitting and guarantee the results are robust.

As mentioned, the feature extraction process involves extracting 125 code features from each EEG segment recorded on a specific channel, where these features are based on various combinations of three rhythmic bases. To address the high dimensionality, feature selection is performed to identify the optimal feature for emotion recognition. To this end, all code features are examined through k-NN, SVM, LDA, or LR, which aimed to evaluate the classification accuracy associated with each channel's code feature so the optimal feature that produces the highest accuracy is identified. As a result, for an EEG system with 32 channels, 32 code features are chosen and denoted as the channel-specific features, each corresponding to the highest accuracy achieved on its respective channel. After that, the best among all channel-specific features is selected. Hence, the single optimal channel-specific feature that generates satisfactory accuracy is determined. In short, the classification method involves initially obtaining channel-specific features by identifying the code feature that provides the highest accuracy on each channel. Then, from the collection of channel-specific features, the optimal feature that demonstrates the most desirable accuracy among all is selected. Consequently, the selection criterion is the accuracy achieved in each emotional case.

## 4. Results and discussion

To achieve the experimental results, MATLAB R2021a is employed for programming and simulating the proposed framework. As for the computation time, it is closely influenced by factors such as MATLAB's parallelization capabilities, cache memories, random access memory (RAM) read/write speed, and other system-specific considerations. In this study, the setup contains an Intel Core i5-10505 central processing unit (CPU) running at 3.20 GHz, 8 GB RAM operating at 2666 MHz, and a 1 TB hard disk drive rotating at 7500 revolutions per min. During the sequencing phase, it costs approximately 18 s for an EEG length of 30 s. For each subject, the extraction of all code features requires approximately 4 s and 6 s when considering 32 channels and 62 channels, respectively. The classification stage, employing k-NN, SVM, LDA, and LR, encompassing both training and testing periods, needs around 27 s per subject. Of note, the algorithm does not impose strict memory requirements, while usually, a larger memory size enhances the speed of simulation runs. Then, the emotion recognition tasks are performed on different test groups so that the single optimal channel-specific feature is found for each group independently. Additionally, the statistical significance is evaluated by operating the ANOVA test, which aimed to demonstrate the suitability of feature selection. Meanwhile, the performance of four classifiers is investigated to determine the most appropriate one for the proposed method. Furthermore, as the optimal channel-specific features contain channel and rhythm details, they are analyzed by assessing the channel locations, rhythmic properties, and individual differences. Finally, a comparative study is conducted to reveal the advantages of the proposed method. More details are presented in the following subsections.

### 4.1. Statistical analysis

**Figure 6:**
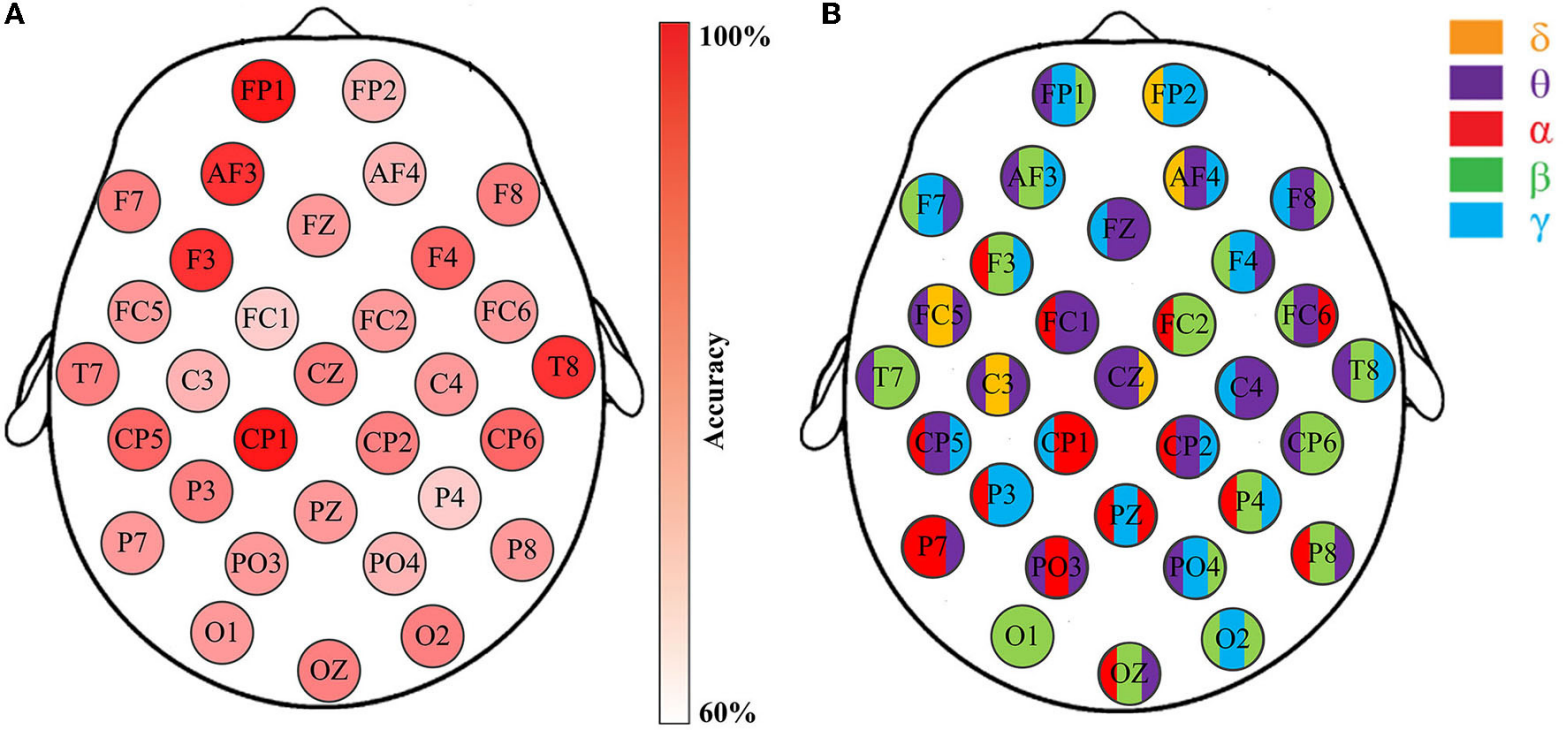
Emotion recognition accuracies through the channel-specific features from respective EEG channels. Data is from DEAP, Subject S8, Group-A (arousal classification): **(A)** The classification accuracies of 32 channels; **(B)** The feature that provides the highest accuracy for the corresponding channels.

In this work, a total of 4000 code features are extracted from each subject of DEAP and MAHNOB, and 7750 code features are extracted from each subject of SEED. Due to the high dimensionality of the data, it is critical to perform feature selection to find the most informative features while discarding those irrelevances. To this end, at the first phase of feature selection, the best code feature that provides the highest accuracy on each EEG channel is selected after training and testing, as shown in Figure 6.

Figure 6 illustrates the effectiveness of the best code feature on each respective channel for the arousal classification of Subject S8, DEAP. The accuracies are depicted in Figure 6A, while Figure 6B highlights the code feature for the corresponding channel. Please note that the SVM classifier with RBF kernel is applied here. Obviously, five distinct rhythms appear on these selected code features with varying classification accuracies. The results indicate that emotion recognition is influenced by channel locations and rhythmic properties. Further analysis reveals that such features generally comprise two or three bases, as opposed to just one kind. For example, Figure 6B demonstrates that apart from the O1 channel with code feature β*ββ*, the rest channels exhibit either two rhythms (e.g., FP2 and FZ) or three rhythms (e.g., FP1 and AF3). More importantly, they imply a connection between rhythms and emotions by considering certain particular appearances in the sequences. For instance, the appearance of θ*γβ* in the FP1 sequence is highly correlated with arousal variations, as it produces the highest accuracy for the arousal classification task. Similar observations can be obtained for others.

**Figure 7:**
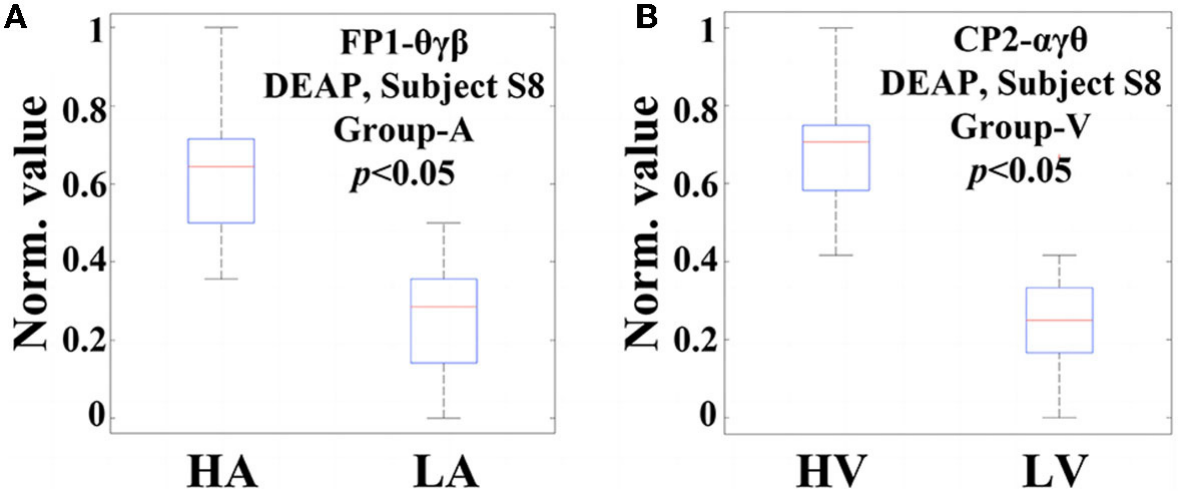
Two examples of ANOVA test box plots for the best code features (channel-specific features) selected on Subject S8, DEAP: **(A)** The code feature θ*γβ* on the FP1 channel for Group-A (arousal classification); **(B)** The code feature α*γθ* on the CP2 channel for Group-V (valence classification).

Now, in order to provide evidence of the statistical significance of the selected code features in relation to emotional states, it is necessary to perform a qualitative analysis. The ANOVA test is a commonly used statistical verification method that involves analyzing differences in group means and variances. When the resulting *p*-value is equal to or smaller than a significance level (typically, 0.05), it can be said that this feature exists a significant difference concerning the particular emotional variation, and is, therefore, suitable for the classification task. Figure 7 displays two examples of ANOVA test box plots for the best code features found on Subject S8, DEAP. In Figure 7A, the code feature θ*γβ* on FP1 for arousal classification is achieved, while Figure 7B tests the code feature α*γθ* on CP2 for valence classification. In this study, both *p*-values are smaller than 0.05, indicating that they possess significant differences in the respective emotional cases. For the arousal classification, higher values of θ*γβ* mostly occur in the HA states, while lower values appear in the LA states, suggesting a positive correlation between arousal level and the appearance of FP1-θ*γβ*. Similarly, for the valence classification, the appearance of CP2-α*γθ* shows an informative clue for indicating the valence level.

As the total size of all code features is huge, several of them may not present statistical significance to emotion recognition. In this regard, the statistical analysis only focuses on those code features selected based on channels, rather than all of the extracted features. Following this way, the ANOVA tests have been performed on the code features selected from each channel for the subjects with the same conditions of DEAP, MAHNOB, and SEED, respectively. The overall results indicate that their *p*-values are all smaller than 0.05, like the two examples illustrated in Figure 7, with similar trends that exhibit statistical significance. It confirms the appropriateness of selecting the best code features through the highest accuracy, which assists the optimal feature selection in the next step.

### 4.2. Single channel-specific feature

**Figure 8:**
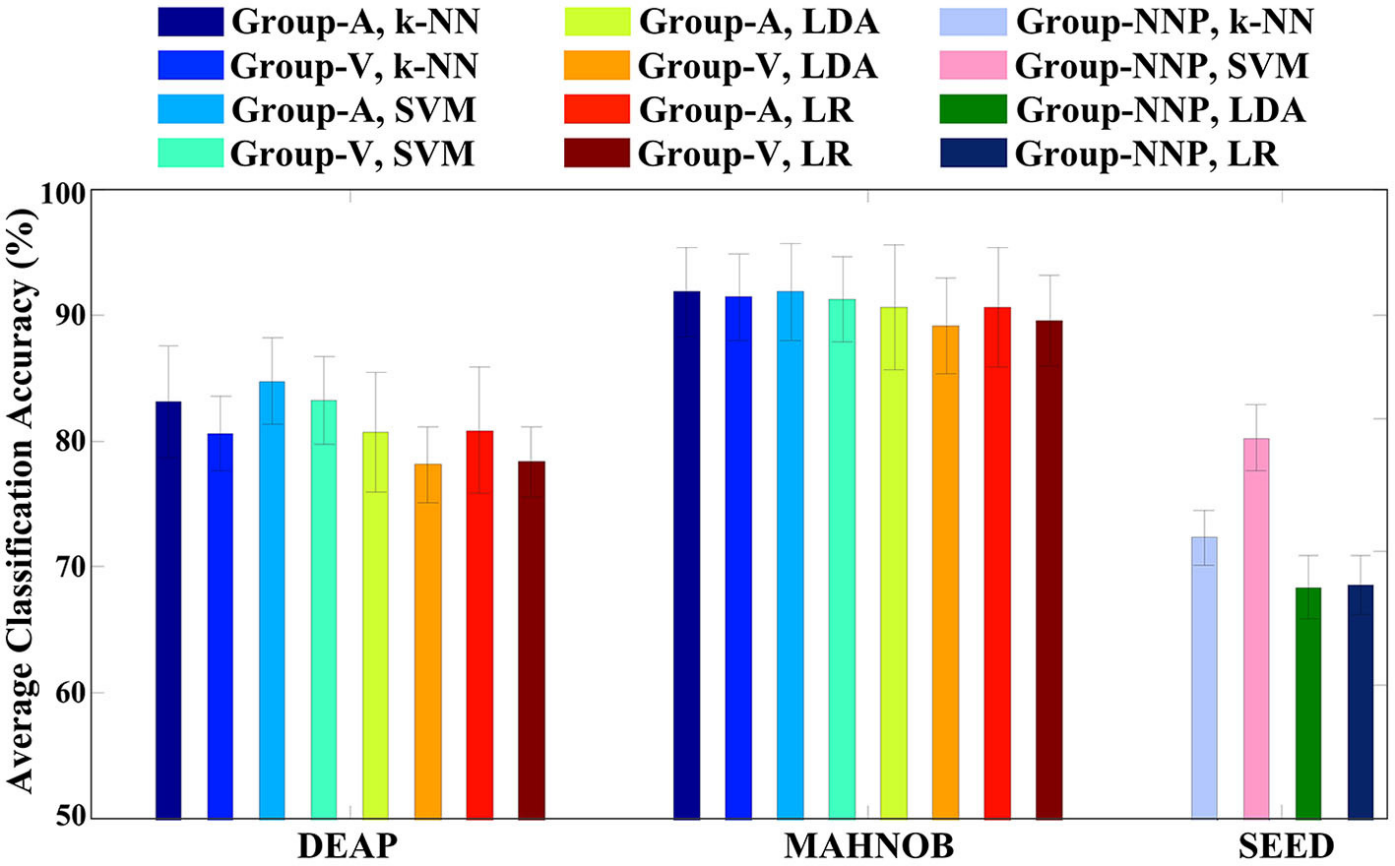
Emotion recognition accuracies on respective test groups of three emotional datasets using the single optimal channel-specific feature selected by the proposed method.

In the previous stage, although those code features are validated to be statistically significant, they may not produce remarkable accuracy as a whole. The variations of accuracies displayed in Figure 6A imply that certain channels (e.g., FP1, AF3, and F3) are more efficient in emotion recognition than others. Additionally, multichannel features can incur issues such as electrode placement discomfort for the subject, longer preparation time, and higher computational costs. Thus, if emotion recognition is implemented through a single feature with satisfactory accuracy, it would greatly benefit the design of portable BCI. To achieve this goal, in the second phase of feature selection, the code features selected on each channel are defined as the channel-specific feature. Subsequently, the best channel-specific feature among 32 (DEAP, MAHNOB) or 62 (SEED) is chosen as the optimal feature accordingly. In this study, cross-corpus evaluations are also performed to investigate the method performances under various conditions. On the DEAP and MAHNOB datasets, the two-classes task is tested, where Group-A includes high arousal and low arousal, and Group-V contains high valence and low valence; on the SEED dataset, Group-NNP (negative, neutral, and positive) is tested. Meanwhile, a histogram comparing the average accuracies of four classifiers is depicted in Figure 8, and the details are summarized in Table 3, where the most impressive result of each case is marked in bold and underlined. Please note that the calculations are the average of all the optimal features that yield the highest accuracy for all subjects in each test group.

**Table 3:** Emotion recognition accuracies (mean ± standard deviation %) using four classifiers.

**Classifier**	**DEAP**		**MAHNOB**		**SEED**
	**Group-A**	**Group-V**	**Group-A**	**Group-V**	**Group-NNP**
SVM	84.77 **±3.44**	83.20 **±4.80**	91.88 **±3.85**	91.67 **±3.51**	78.52 **±2.47**
k-NN	83.13 ± 4.40	80.55 ± 2.96	91.46 ± 4.29	91.25 ± 3.69	61.04 ± 2.03
LDA	80.70 ± 4.80	78.13 ± 2.98	90.63 ± 4.96	89.17 ± 3.81	57.33 ± 2.41
LR	80.86 ± 5.02	78.36 ± 2.81	90.00 ± 5.11	89.58 ± 3.59	57.48 ± 2.20

The best classification results are marked in bold and underlined.

In Table 3, SVM outperforms the other three classifiers with the highest overall performances, especially on Group-NNP, suggesting that SVM achieves a stable performance even with an increase in the number of classes. That may be due to the properties of the classifiers. First, LDA and LR contain the assumptions of multivariate normality, with LDA being more efficient in parameter estimation by utilizing more information about the data, while LR relies on fewer assumptions and is more robust to non-Gaussian data (Decruyenaere et al., 2015). In practice, they often produce similar results, which is why their accuracies are similar in Table 3. Second, k-NN is a cluster-based classifier that considers a group of neighbors around the testing data for classification. Hence, it is sensitive to the scale of the data, revealing its accuracy closely decided by the quality of the training data. In this regard, for the high-dimensional cases with multiple classes (e.g., Group-NNP), k-NN may result in wrong predictions due to its difficulties in calculating the distance in each class. Finally, SVM and its variants create a hyperplane with a specific margin to split the training data into distinct categories. It performs well when there is a large gap between the classes and maintains good results in high-dimensional cases. Such results also indicate that the distribution of the optimal feature may be well-suited to SVM. Consequently, this investigation demonstrates that SVM is an appropriate machine learning classifier in this study and helpfully implies the efficiency of the proposed method under various conditions as it generates satisfactory accuracies with a single feature only.

After discussing the classifiers, the investigation concentrates on the optimal channel-specific features obtained from different subjects using SVM. In this study, to study their associated channel locations, the scalp is further divided into five regions (frontal, central, parietal, temporal, and occipital). A statistical analysis is then conducted by considering the prominent regions, as shown in Table 4.

**Table 4:** Statistical percentages of optimal channel-specific features in terms of scalp region.

**Scalp region**	**DEAP**		**MAHNOB**		**SEED**
	**Group-A**	**Group-V**	**Group-A**	**Group-V**	**Group-NNP**
Frontal	37.50%	43.75%	45.83%	37.50%	53.33%
Central	12.50%	12.50%	12.50%	8.33%	20.00%
Parietal	21.88%	28.13%	29.17%	8.33%	26.67%
Temporal	12.50%	9.38%	8.33%	33.33%	0.00%
Occipital	15.63%	6.25%	4.17%	12.50%	0.00%

The maximums are marked in bold and underlined.

The results of Table 4 demonstrate that the optimal features of different subjects are prominently located in the frontal region, which regulates high-level cognitive capabilities, including emotion, judgment, language, and memory (Mansouri et al., 2017). Generally, the frontal region is viewed as the control panel of personality and expression ability, and as emotion is the cognitive awareness from stimuli, it is persuasive evidence to exhibit a relationship with the frontal region. Therefore, this investigation validates that the proposed method can properly select the single optimal channel-specific feature in each emotional scenario.

Next, the assessments of rhythmic properties are performed. In this study, as the proposed features are inspired by the genetic code consisting of three bases, they enclose any three of five brain rhythms, so their combinations contain three cases: one rhythm (e.g., β*ββ* and α*αα*), two rhythms (e.g., δ*θθ* and α*γα*), and three rhythms (e.g., α*γθ* and θ*γβ*). In this regard, the number of rhythmic types that occur in the optimal channel-specific features found from all subjects is analyzed, which aimed to explore the rhythmic patterns in terms of temporal dynamics during emotional variations. The results are listed in Table 5.

**Table 5:** Statistical percentages of optimal channel-specific features in terms of rhythmic types.

**Number of rhythmic types**	**DEAP**		**MAHNOB**		**SEED**
	**Group-A**	**Group-V**	**Group-A**	**Group-V**	**Group-NNP**
One	0.00%	0.00%	0.00%	4.17%	0.00%
Two	50.00%	46.88%	37.50%	50.00%	33.33%
Three	50.00%	53.13%	62.50%	45.83%	66.67%

The maximums are marked in bold and underlined.

Table 5 reveals that the optimal feature primarily includes two or three rhythms, suggesting that the involvement of multiple kinds may enhance the reliability of emotion recognition. This finding discloses the importance of identifying vital rhythmic combinations, as one rhythm alone may not be sufficient for classification. Additionally, the results indicate that the changes within the rhythm sequences tend to contain valuable information, while constant data may not be as significant for the proposed code features. As mentioned, all five rhythms have been linked to emotions, so focusing on the variations of two or three specific rhythms within the sequence data helps to assess emotion, as enabled by the proposed method. In short, from the method evaluation, the classification accuracies are impressive, demonstrating the feature derived from a particular combination of five brain rhythms with a size of three bases is beneficial for EEG-based emotion recognition.

**Figure 9:**
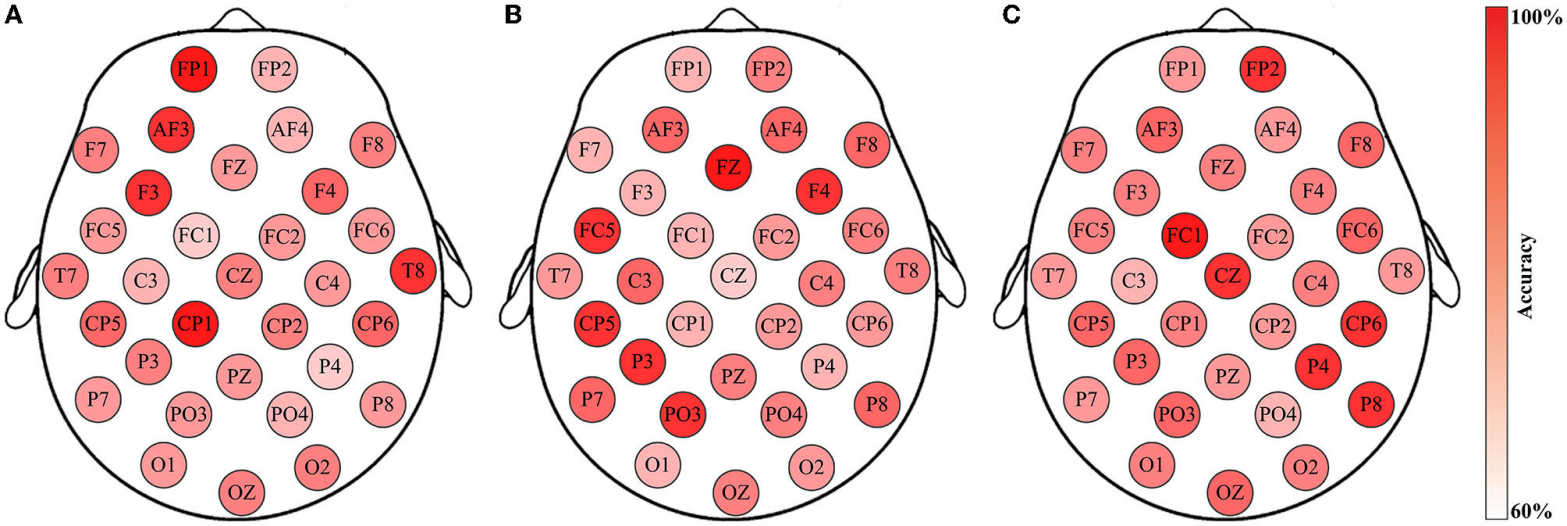
Emotion recognition accuracies using the channel-specific features for Group-A (arousal classification) of the three subjects from the DEAP dataset: **(A)** Subject S8; **(B)** Subject S17; **(C)** Subject S29. Such findings reveal individual differences in EEG-based emotion recognition.

Furthermore, an insightful observation regarding the individual differences in emotion recognition is achieved by considering the classification accuracies between different subjects in the same test group. A result generated by three subjects in Group-A of the DEAP dataset is illustrated in Figure 9. Although all of the channel-specific features are extracted in the same manner, and the classifications are all implemented by SVM, the method performances are diverse across subjects. Meanwhile, as the channel-specific features are dependent on the channels and subjects, it is not easy to conclude their specific types among all cases. For example, in Figure 9A, the optimal feature of Subject S8 is in the FP1 channel, but it improperly generates reliable accuracies for the other two subjects. In addition, the results presented in Figure 9A are the same as Figure 6A, where the arousal classification accuracies of Subject S8 in DEAP are drawn. Then, Figure 6B depicts the rhythmic features that provide the highest accuracy for the corresponding channels, i.e., the channel-specific features. Based on that, what rhythmic feature is valuable to the respective channel can be answered, such as FP1 is θ*γβ*, AF3 is θ*βγ*, and F3 is α*βγ*. Concerning the subjects of Figure 9, similar to the variations of classification accuracies, their corresponding rhythmic features also vary a lot, like in Figure 6B, so the rhythmic features on each respective channel are with any three of the five brain rhythms, while the details are unsettled individually. In this regard, the results indicate that the FP1 channel-specific feature is only vital for Subject S8, revealing the subject-dependent effect of emotion recognition. Such individual differences are consistent with the previous study (Lim, 2016), as emotion is a subjective behavior influenced by cultural, experiential, and background factors. Consequently, the proposed method of selecting the single optimal channel-specific feature based on subject-dependent effect is meaningful for emotion recognition through minimal data.

### 4.3. Comparative study

For a comprehensive comparison, several scenarios with various conditions have been assessed. First, the experiments using all features based on the majority voting method are performed. For instance, when using the k-NN classifier with LOTO-CV, each of the extracted features can generate the classification result, and the results from all features are collected (i.e., 4000 results on each case of DEAP and MAHNOB, and 7750 results on each case of SEED). Accordingly, the class that occurs most frequently among those results from the same case is selected as the final classification result. Based on that, the emotion recognition accuracies through all features employing four classifiers are obtained. Such comparative analysis reveals that the proposed solution of utilizing the single optimal channel-specific feature outperforms the approach that considers all features, as the overall accuracies achieved from the optimal feature approach are higher than the all features approach, approximately 13%−35%. It can be inferred that not all scalp channels are intensely associated with emotion, which might explain the relatively lower performance when combining insignificant features in the majority voting method. The inclusion of these less relevant features could potentially impact the classification. Hence, the proposed method emphasizes the optimal feature that yields the highest accuracy, rather than incorporating all available features.

Second, a comparative study with the previous studies is summarized in Table 6, where the best results are marked in bold and underlined. Compared to deep learning techniques, such as deep belief networks (DBNs) (Zheng and Lu, 2015), long short-term memory (LSTM) (Sharma et al., 2020), and 4D-ANN (Xiao et al., 2022), the classification accuracies employing the proposed method are not the most remarkable, while it accomplishes impressive performances by utilizing a single channel-specific feature, indicating that only one electrode is needed to collect the corresponding signals for emotion recognition. In contrast, the neural network architecture usually requires more channels and features, as a vast amount of data is significant to acquire informative features that produce superior accuracies. However, when fewer channels are used, as in the case of portable BCI equipped with limited channels, training a network that maintains high remarkable classification accuracies is challenging. Hence, the main advantage is that the proposed method overcomes such a limitation by employing minimal data while providing satisfactory accuracy, making it a potential solution that is compatible with portable scenarios. Moreover, the cross-corpus results indicate its availability across datasets, including dimensional emotions (arousal and valence) and discrete emotions (negative, neutral, and positive). This comparison also highlights the potential of the proposed method to be a general solution for emotion recognition, as interpreting EEG signals in a sequential format and extracting brain rhythm code features based on three bases leads to improve EEG-based emotion recognition through fewer sources.

**Table 6:** Comparative study with the previous studies.

**Work**	**Channels**	**Classifier**	**Method/Feature**	**Classification accuracy (%)**				
				**DEAP**		**MAHNOB**		**SEED**
				**Group-A**	**Group-V**	**Group-A**	**Group-V**	**Group-NNP**
Menezes et al. (2017)	4	SVM	Statistics, HOC, PSD	67.10	88.90	/	/	/
Javidan et al. (2021)	2	SVR	MSCE	/	67.45	/	/	/
Kannadasan et al. (2023)	11, 13	SVM	DEFS	74.23	73.60	/	/	/
Sharma et al. (2020)	32	LSTM	Statistics, PSO, ToC	85.21	84.16	/	/	/
Anuragi et al. (2022)	10	Ensemble bagged tree	FBSE-EWT, NCA	84.30	83.90	/	/	78.10
Xiao et al. (2022)	32, 62	4D-ANN	Spatial-spectral-temporal representations	97.39	96.90	/	/	96.25
Zheng and Lu (2015)	4	DBNs	DE, asymmetry	/	/	/	/	82.88
Huang et al. (2019)	14	SVM	PSD, weight function	/	/	71.88	66.25	/
Wang et al. (2022)	32	HSLT	Discriminative spatial information	65.75	66.51	66.20	66.63	/
Piho and Tjahjadi (2018)	32	k-NN	Statistics, adaptive windowing	89.84	89.61	94.00	94.60	/
This study	1	SVM	Code feature, BRS	84.77	83.20	91.88	91.67	78.52

The best classification results are marked in bold and underlined.

Finally, in the previous study (Li et al., 2019), the seizure detection task using the BRS technique has been proposed. Although the EEG data are processed by BRS, the main objective and the classification method are entirely different, as the EEG recordings exhibit distinct properties in the two cases. Previously, the sequence characterization by adding indices for enriching the seizure-related characteristics toward the detection task has been considered. In this study, the code features are extracted from the rhythm sequences, and conventional classifiers are employed. Then, based on the accuracies, the single optimal channel-specific feature is selected for emotion recognition. In short, both studies reveal the benefits of the BRS, which not only extends the understanding of brain rhythms but also presents a promising approach for classifying EEG signals in various neuroscience applications, such as seizure detection and emotion recognition.

## 5. Conclusion

The code features extracted from the sequence generated by the BRS technique have been proposed in this study. Compared to the previous study, the proposed method is adequate to provide a potential solution for designing the portable emotion-aware BCI device, as the single optimal channel-specific feature of each case can be efficiently selected for the subjects from three emotional datasets, yielding classification accuracies approximately 83–92% for two-classes task and approximately 78% for three-classes task. Such results are impressive when operating minimal data for emotion recognition. Meanwhile, the results reveal that the SVM classifier is proper to the proposed features. Further investigations of such optimal features disclose that their channel locations are mainly on the frontal region, and the rhythmic properties are typical with either two or three kinds in the combinations. Finally, an insightful observation demonstrates that emotion recognition exhibits individual differences. Therefore, to accomplish satisfactory classification accuracy by employing a single feature, the subject-dependent effect should be analyzed, which can be acquired using the proposed method. In future, to apply it in the practical BCI system, the hardware that implements the optimal channel-specific feature and SVM is the main task. In addition, to further enhance the classification accuracy, with the help of sufficiently large feature size, recent advanced approaches, such as self-attention enhanced deep residual network (Xie et al., 2023), multiscale superpixel-wise prophet model (Ma et al., 2023), and multistage stepwise discrimination (Chen et al., 2023), will be developed and investigated.

## Data availability statement

The original contributions presented in the study are included in the article/supplementary material, further inquiries can be directed to the corresponding authors.

## Author contributions

JWL and DL designed the method and analyzed the experimental data with the support of YC, JJL, and RJC. JWL wrote the first draft of the manuscript with the support of DL, LJW, and XXZ. JCR, HMZ, and XL directed the manuscript. All authors participated in the scientific discussion, contributed to the manuscript, and approved the submitted version.
